# Investigating the Thioredoxin and Glutathione Systems’ Response in Lymphoma Cells after Treatment with [Au(d2pype)2]Cl

**DOI:** 10.3390/antiox10010104

**Published:** 2021-01-13

**Authors:** Sicong Wang, Yaoying Lu, Kyra Woods, Giovanna Di Trapani, Kathryn F. Tonissen

**Affiliations:** 1 School of Environment and Science, Griffith University, Nathan, Brisbane, QLD 4111, Australia; sicong.wang@griffith.edu.au (S.W.); yaoying.lu@griffithuni.edu.au (Y.L.); k.woods@uq.edu.au (K.W.); 2 Griffith Institute for Drug Discovery, Griffith University, Nathan, Brisbane, QLD 4111, Australia; 3 School of Chemistry and Molecular Biosciences, The University of Queensland, St Lucia, Brisbane, QLD 4072, Australia

**Keywords:** lymphoma, thioredoxin, glutathione, ROS, apoptosis, gold-based compounds

## Abstract

Lymphoma is a blood cancer comprising various subtypes. Although effective therapies are available, some patients fail to respond to treatment and can suffer from side effects. Antioxidant systems, especially the thioredoxin (Trx) and glutathione (GSH) systems, are known to enhance cancer cell survival, with thioredoxin reductase (TrxR) recently reported as a potential anticancer target. Since the GSH system can compensate for some Trx system functions, we investigated its response in three lymphoma cell lines after inhibiting TrxR activity with [Au(d2pype)2]Cl, a known TrxR inhibitor. [Au(d2pype)2]Cl increased intracellular reactive oxygen species (ROS) levels and induced caspase-3 activity leading to cell apoptosis through inhibiting both TrxR and glutathione peroxidase (Gpx) activity. Expression of the tumour suppresser gene TXNIP increased, while GPX1 and GPX4 expression, which are related to poor prognosis of lymphoma patients, decreased. Unlike SUDHL2 and SUDHL4 cells, which exhibited a decreased GSH/GSSG ratio after treatment, in KMH2 cells the ratio remained unchanged, while glutathione reductase and glutaredoxin expression increased. Since KMH2 cells were less sensitive to treatment with [Au(d2pype)2]Cl, the GSH system may play a role in protecting cells from apoptosis after TrxR inhibition. Overall, our study demonstrates that inhibition of TrxR represents a valid therapeutic approach for lymphoma.

## 1. Introduction

Lymphoma is a type of cancer that develops in the lymphatic system. It can be divided into two subtypes: Hodgkin’s (HL) and non-Hodgkin’s lymphoma (NHL). It is reported that NHL generally has a more rapid cell growth than HL [1]. Hodgkin’s lymphoma is most commonly diagnosed in young people and adults over 55 [2]. Non-Hodgkin’s lymphoma is the fifth most common cancer type in developed countries [3]. ABVD (Doxorubicin hydrochloride, bleomycin, vinblastine sulfate, and dacarbazine) [4] and CHOP-R (rituximab plus cyclophosphamide, doxorubicin, vincristine and prednisone) [5] are two standard chemotherapy regimens to treat Hodgkin’s and non-Hodgkin’s lymphoma, respectively. However, only 50–60% of lymphoma patients achieve long-term survival rates. In addition, some surviving patients still suffer from the side effects of chemotherapy, including cardiac damage [6]. Therefore, novel therapeutic strategies to treat lymphoma are needed.

The abnormal metabolism in various cancers causes oxidative stress, a recognized biomarker of cancer [7,8,9,10]. Cancer cells adapt to this condition by upregulating antioxidant molecules to protect themselves from death resulting from increased levels of reactive oxygen species (ROS). However, the ROS levels in cancer cells remain close to the maximum cytotoxicity threshold [11]. Therefore, when the antioxidant capability is disrupted by inhibitors, the redox balance in cancer cells with oxidative stress is more easily disrupted and pushed to maximum cytotoxicity threshold when compared with healthy cells [12].

The thioredoxin (Trx) and glutathione (GSH) systems are two cellular antioxidant systems that regulate redox homeostasis by counteracting the increased ROS production in cells [13,14]. The Trx system is made up of thioredoxin (Trx), thioredoxin reductase (TrxR), and NADPH. The GSH system consists of glutathione (GSH), glutathione reductase (GR), glutathione peroxidase (GPx), glutaredoxin (Grx), glutathione S-transferases (GST) and NADPH [15]. Additionally, these two systems can also regulate several signalling pathways through reversible disulfide bond formation in the target proteins, especially the Trx system [16]. Moreover, the Trx and GSH systems can also regulate cellular behaviour via S-nitrosylation or persulfidation of target proteins [17,18]. The active form of Trx reduces disulfide bonds in target proteins, and becomes oxidized itself, then the oxidized form of Trx can be reduced by TrxR to return to the active reduced form [19]. The expression of the Trx system proteins is upregulated in cancers, leading to cancer cell proliferation and survival [20]. Studies have shown that the inhibition of TrxR activity can result in cancer cell apoptosis [21,22].

Gold-based molecules have been shown to have anticancer ability by interacting with the selenocysteine and cysteine residues in proteins [23,24]. In addition, a study showed that gold (I)-based compounds have the selectivity of preferentially inhibiting selenocysteine amino acid residues [25]. Since the activity of TrxR depends on the selenocysteine residue in its active site, gold (I)-based molecules can readily target TrxR and inhibit its activity. Auranofin is a linear gold(I) phosphine compound, which has been shown to have anticancer activity by inhibiting TrxR activity [26,27]. However, studies also reported that auranofin can inhibit Trx activity in cells through the inhibition of TrxR [28]. A gold(I) phosphine TrxR inhibitor, bis-chelated tetrahedral gold(I) phosphine complex [Au(d2pype)2]Cl was designed to target TrxR [24,29]. Studies have indicated that [Au(d2pype)2]Cl has a higher selectivity towards TrxR than auranofin [29], and recently has been shown to exhibit anticancer effects against multiple myeloma (MM) and chronic myeloid leukaemia (CML) [30,31]. However, it has not yet been tested against lymphoma. Moreover, the effect of [Au(d2pype)2]Cl towards the GSH system has not been previously assessed in any cancer cell lines. In this study, a classic Hodgkin’s lymphoma cell line (KMH2) and two non-Hodgkin’s lymphoma cell lines, an activated B-cell diffuse large B-cell lymphoma (ABC-DLBCL) cell line (SUDHL2) and a germinal centre B-cell diffuse large B-cell lymphoma (GCB-DLBCL) cell line (SUDHL4), will be used as a model to assess the anticancer activity of [Au(d2pype)2]Cl and investigate the Trx and GSH systems’ response after treatment with [Au(d2pype)2]Cl.

## 2. Materials and Methods

### 2.1. Cells and Reagents

Three human lymphoma cell lines (KMH2, SUDHL2 and SUDHL4) were gifted by Professor Maher Gandhi (Princess Alexandra Hospital, Brisbane, Australia). They were authenticated by the Griffith University DNA Sequencing Facility (GUDSF) using the STR profiling method (GenePrint^®^ 10 System, Promega, Madison, WI, USA). Cells were cultured in RPMI-1640 medium (Gibco, Gaithersburg, MD, USA) containing 10% (*v/v*) foetal bovine serum (FBS) (Bovagen, France), 200 mM of L-glutamine, 100 U/mL of penicillin and 100 μg/mL streptomycin (Gibco, Gaithersburg, MD, USA). [Au(d2pype)2]Cl was gifted by Sue Berners-Price (Glycomics, Griffith University, QLD, Australia). Auranofin was purchased from Cayman Chemicals (Ann Arbor, MI, USA), and sodium selenite, and buthionine sulfoximine (BSO) were obtained from Sigma Chemicals (Sydney, NSW, Australia). RT-qPCR oligonucleotides were purchased from Integrated DNA Technologies (IDT, Singapore). The β-tubulin polyclonal antibody (cat no. ab6046) was obtained from Abcam (Cambridge, UK).

### 2.2. Gold Compound Preparation

Auranofin was dissolved in DMSO to a 10 mM stock concentration. [Au(d2pype)2]Cl was dissolved in ethanol to a 300 μM stock concentration. Gold compounds were diluted to the desired concentrations in either 1× PBS or phenol red-free RPMI-1640 medium before each experiment. The structure of auranofin and [Au(d2pype)2]Cl has been previously published [30].

### 2.3. Cell Proliferation Assay

Cells were seeded (40,000 cells per well) into a 96-well plate (Corning, NY, USA). The vehicle control was 0.1% (*v/v*) ethanol in RPMI-1640 phenol red free media without cells. Cells were incubated at 37 °C for 24 h and 48 h. Then 10 µL of filtered sterile 5 mg/mL MTT was added to each well and incubated at 37 °C for 3 h. Following this, 25 µL of 20% (*w/v*) SDS/0.01 M HCl was added to each well and incubated overnight at 37 °C. The plate was read at 570 nm in the FLUOstar Omega plate reader (BMG Lab-tech, Ortenberg, Germany) the next day.

### 2.4. TrxR Activity Assay

TrxR activity assays were performed as described previously [30]. Briefly, TrxR activity was measured in a SpectraMax M3 plate reader (Molecular Devices, VIC, Australia) based on the NADPH-dependent reduction of DTNB. Cells were lysed in 0.5% (*v/v*) Nonidet P-40 lysis buffer (150 mM NaCl, 50 mM Tris-Cl, 0.5% (*v/v*) Nonidet P-40, 0.5 mM EDTA pH 8, 2 mM PMSF and 1 μL/mL protease inhibitor cocktail VI (Astral Scientific, Sydney, NSW, Australia)). Cells lysates were treated with or without 8 μM of auranofin at room temperature for 30 min to remove non-TrxR-specific DTNB reduction. The TrxR activity was determined using a buffer with 125 mM potassium phosphate pH 7.5, 2.5 mM EDTA, 0.25 mM NADPH, and 3.125 mM DTNB. TNB production was measured at 412 nm for 10 min. The specific TrxR activity (mU/mg protein) was calculated by normalizing the units of TrxR activity with the content of protein in each sample.

### 2.5. GR Activity Assay

GR activity assays were performed as described previously [32]. Briefly, GR activity was determined in a SpectraMax M3 plate reader (Molecular Devices, VIC, Australia) based on the NADPH-dependent reduction of GSSG by GR. Cells were lysed in 0.5% (*v*/*v*) Nonidet P-40 lysis buffer (150 mM NaCl, 50 mM Tris-Cl, 0.5% (*v*/*v*) Nonidet P-40, 0.5 mM EDTA, pH 8, 1× PBS, 2 mM PMSF and 1 μL/mL protease inhibitor cocktail VI (Astral Scientific, Sydney, NSW, Australia)). The GR activity was measured using a buffer with 125 mM potassium phosphate pH 7.5, 2.5 mM EDTA, and 0.25 mM NADPH. NADPH oxidation was measured at 340 nm for 10 min. The specific GR activity (mU/mg protein) was calculated by normalizing the units of GR activity with the content of protein in each sample.

### 2.6. Measurement of GSH and GSSG

The measurement of GSH and GSSG was determined as described previously [33,34] with modification. Cells were lysed in ice cold 1% (*w/v*) sulfosalicylic acid (SSA) extraction buffer containing 0.1% (*v*/*v*) Triton X-100 and sonicated at 20 kHz for 20 s. Samples were centrifuged at 3000× *g* for 4 min at 4 ℃. The supernatant was transferred into new tubes.

Total GSH was measured in a SpectraMax M3 plate reader (Molecular Devices, Melbourne, VIC, Australia) based on the NADPH-dependent reduction of DTNB by GSH. The GSH was measured using a buffer with 0.25 mM NADPH, 0.5 mM DTNB, and 0.2 U GR. TNB production was determined at 412 nm for 5 min. The total GSH concentration was calculate from a GSH standard curve.

GSSG measurement was determined using 2-vinylpyridine as masking agent for reduced GSH. Briefly, samples were treated with 2% (*v/v*) 2-vinylpyridine and incubated for 1 h. Then 0.3 M triethanolamine was added and incubated for 10 min. The GSSG was measured using a buffer with 0.25 mM NADPH, 0.5 mM DTNB, and 0.2 U GR. TNB production was determined at 412 nm for 5 min. The total GSSG concentration was calculated from a GSSG standard curve.

The GSH/GSSG ratio was calculated using the following formula:

GSH/GSSG = reduced GSH (Total GSH − 2 × GSSG)/GSSG(1)

### 2.7. Gpx Activity Assay

Gpx activity assays were performed as described previously [35]. Briefly, Gpx activity was measured in a SpectraMax M3 plate reader (Molecular Devices, VIC, Australia) based on the NADPH-dependent reduction of GSSG by GR. Cells were lysed in 0.5% (*v/v*) Nonidet P-40 lysis buffer (150 mM NaCl, 50 mM Tris-Cl, 0.5% (*v/v*) Nonidet P-40, 0.5 mM EDTA, pH 8, 1X PBS, 2 mM PMSF and 1 μL/mL protease inhibitor cocktail VI (Astral Scientific, Sydney, NSW, Australia)). The Gpx activity was measured using a buffer with 125 mM potassium phosphate pH 7.5, 2.5 mM EDTA, 0.25 mM NADPH, 1.25 mM sodium azide, 2.5 mM GSH, 5U GR, 0.125 mM t-BOOH. NADPH oxidation was measured at 340 nm for 10 min. The specific Gpx activity (mU/mg protein) was calculated by normalizing the units of Gpx activity with the content of protein in each sample.

### 2.8. Intracellular ROS Measurement Assay

The ROS level was determined using H_2_DCFDA as described previously [36]. Briefly, lymphoma cells were treated with inhibitors for 24 h before incubation with 5 μM H_2_DCFDA (Molecular probes, CA, USA) for 30 min. DCF, the oxidized form of H_2_DCFDA, was measured by using the FLUOstar Optima plate reader (BMG Lab-tech, Ortenberg, Germany) with a fluorescence excitation wavelength of 495 nm and emission wavelength of 515 nm. The results were normalized to cell number to achieve relative ROS levels.

### 2.9. Caspase-3 Activity Assay

The determination of caspase activity in untreated and treated lymphoma cells was determined using Ac-DEVD-AMC (Cayman Chemical Company, Ann Arbor, MI, USA) as described previously [37]. Briefly, cells were seeded into 24-well plate and treated with chemical drugs for 24 h. Cells were washed twice with 1× PBS and resuspended in 20 µL of 1× PBS. Cell suspension was added into a black clear-bottomed 96-well plate with adding 80 µL of caspase-3 buffer (5 mM DTT; 100 mM HEPES, pH 7.5; 10% (*w/v*) Sucrose; 0.1% (*v/v*) Nonidet P-40; 50 µM Ac-DEVD-AMC (Cayman Chemical Company, Ann Arbor, MI, USA)). The plate was immediately incubated in the FLUOstar Omega plate reader (BMG Labtech, Ortenberg, Germany) for 15 min at 37 ℃, and AMC was determined with a fluorescence excitation wavelength of 370 nm and emission wavelength of 445 nm.

### 2.10. Reverse Transcriptase-Quantitative PCR (RT-qPCR)

Total RNA was extracted from KMH2, SUDHL2, and SUDHL4 lymphoma cells using TRIsure™ Total RNA Lysis solution (Bioline, Sydney, NSW, Australia) as per manufacturer’s guidelines. cDNA was synthesized by total RNA using the GoScript™ Reverse Transcription Mix (Promega, Madison, WI, USA). RT-qPCR was performed using cDNA by SensiFAST™ SYBR^®^ No-Rox Kit (Bioline, Sydney, NSW, Australia). The RT-qPCR oligonucleotides (Integrated DNA Technologies, Singapore) are listed in Table 1.

The following reaction conditions were used: 95 °C for 2 min followed by 40 cycles of 95 °C for 10 s, 60 °C for 15 s and 72 °C for 20 s. Quantification was performed on Bio-Rad CFX96 Real-Time PCR Detection System (Bio-Rad, Hercules, CA, USA) based on the manufacturer’s instructions. The relative mRNA expression was measured by using the comparative cycle threshold algorithm (∆∆Ct) method. The mRNA expression levels were normalized against the expression levels of ribosomal protein L32 (RPL32) [38].

### 2.11. Western Blot Analysis

Samples were loaded on 10% SDS/polyacrylamide gel and electrophoresed. Then proteins were transferred onto polyvinylidene difluoride (PVDF) membrane using the Tran-Blot Turbo system (Bio-Rad, Hercules, CA, USA) and detected by anti-PARP1 (Cell Signalling, Davers, MA, USA) and anti- β-tubulin (Abcam, Cambridge, UK). Appropriate secondary antibodies were used, and then the signal detection was enhanced by chemiluminescence (ECL) kit (GE Healthcare, Chicago, IL, USA).

### 2.12. Statistical Analysis

All values are displayed as mean ± SEM. Data in this paper were analysed by the Graphpad Prism 8 software (GraphPad, San Diego, CA, USA). Statistical significance was obtained by the specified statistical test. *p* < 0.05 was considered significant.

## 3. Results

### 3.1. Lymphoma Cells Have Up-Regulated Antioxidant Systems

To examine whether lymphoma cells have increased antioxidant capability, the Brune lymphoma mRNA expression dataset [39] was analysed. The results showed that several antioxidant genes are upregulated in HL and DLBCL patient samples (Table 2). It is notable that the mRNA expression levels of TXN, GPX1, GPX4, and GLRX2 were ranked in the top 1% upregulated genes in both HL and DLBCL when compared with healthy cells. In addition, the expression of GLRX3 in DLBCL also ranks within the top 1% of upregulated genes. Although expression of those genes was all upregulated in lymphoma, they have different fold changes between HL and DLBCL. The expression of TXN in HL was higher than that observed in DLBCL, while the expression of GPX1, GPX4, and GLRX3 in HL was lower than that in DLBCL. However, not all antioxidant genes exhibit increased expression in the two types of lymphoma, indicating that the upregulated antioxidants may play a specific role in lymphoma development or progression. The upregulated antioxidants represent crucial proteins that are important for the Trx and GSH systems [16,17,18] and therefore warranted further study.

### 3.2. [Au(d2pype)2]Cl Inhibits Cell Proliferation in Lymphoma Cell Lines

**Figure 1:**
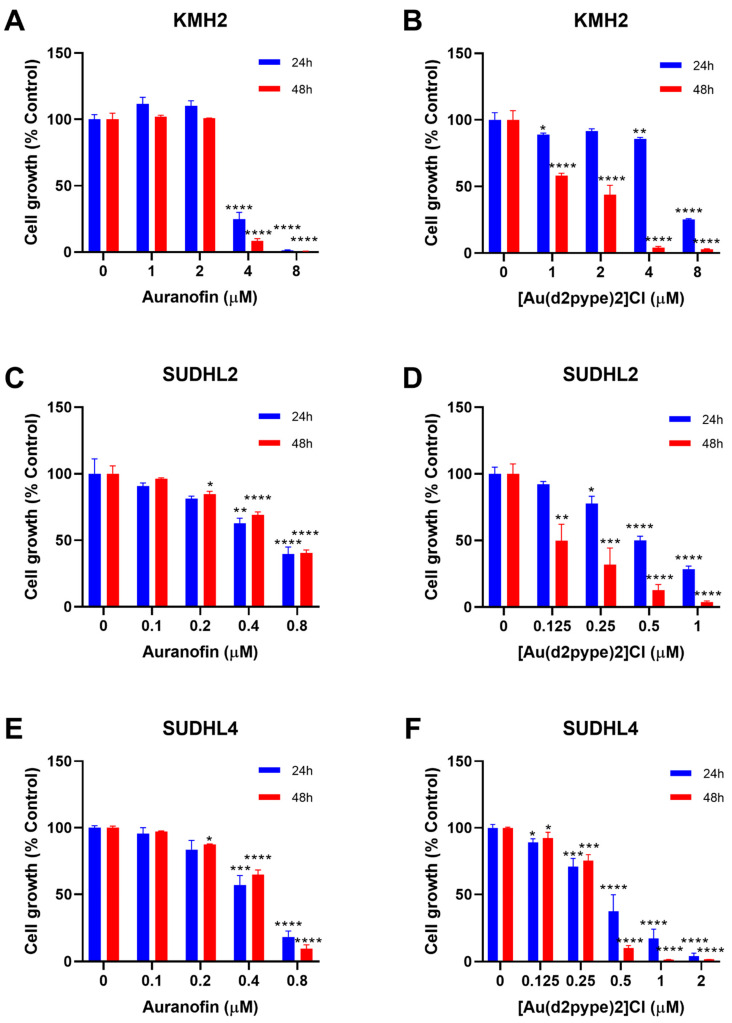
[Au(d2pype)2]Cl and auranofin inhibit lymphoma cell growth in lymphoma cells. (**A**) KMH2, (**C**) SUDHL2, and (**E**) SUDHL4 cells were treated with indicated concentrations of Auranofin for 24 and 48 h. (**B**) KMH2, (**D**) SUDHL2, and (**F**) SUDHL4 cells were treated with different concentrations of [Au(d2pype)2]Cl for 24 and 48 h. Cell growth was detected by MTT assay. Treated cells were compared with the untreated cells. Data were analysed by one-way ANOVA using Dunnett’s post-test. Values indicate mean ± SEM (*n* = 3). * *p* < 0.05, ** *p* < 0.01, *** *p* < 0.001, **** *p* < 0.0001.

Previous studies have shown that cancer cell treatment with auranofin resulted in cell proliferation inhibition and cell death in MM [21]. Therefore, auranofin and [Au(d2pype)2]Cl, which also inhibits TrxR activity [26,29], were used to determine their effect on lymphoma cell proliferation. MTT assays were carried out to determine the cell proliferation of three lymphoma cell lines, KMH2, SUDHL2, and SUDHL4 cells that are representatives of HL, ABC-DLBCL, and GCB-DLBCL respectively. The results showed that with an increasing concentration of auranofin and [Au(d2pype)2]Cl, cell proliferation was decreased in all three lymphoma cell lines (Figure 1).

The auranofin treatment group showed that cell proliferation inhibition was similar after 24 and 48 h treatment (Figure 1A,C,E). In addition, the 24 and 48 h treatment had a statistically significant decrease at the same concentration of auranofin in each cell line. KMH2 cells showed a statistically significant decrease at 4 μM after 24 and 48 h treatment (Figure 1A). SUDHL2 and SUDHL4 cell lines were more sensitive to auranofin, showing a significant statistical decrease at 0.4 μM auranofin after 24 h treatment. After 48 h treatment, a significant statistical decrease at 0.2 µM auranofin was shown in both SUDHL2 and SUDHL4 cell lines (Figure 1C,E).

In the [Au(d2pype)2]Cl treatment group, treatment for 48 h showed a more significant cell proliferation inhibition when compared with treatment for 24 h for all cell lines (Figure 1B,D,F). In KMH2 cells, a statistically significant decrease was shown at 4 μM [Au(d2pype)2]Cl after 24 h treatment, while a statistically significant decrease was achieved at 1 μM Au-SBP after 48 h treatment (Figure 1B). Although SUDHL2 cells showed a significant decrease at a concentration of 0.25 μM for both 24 and 48 h treatment, the level of cell proliferation inhibition after 48 h treatment was almost two times greater than that observed after 24 h treatment (Figure 1D). For SUDHL4 cells, a low concentration of [Au(d2pype)2]Cl (below 0.5 μM) showed similar inhibition effectiveness between 24 and 48 h treatment, while the 48 h treatment showed a higher inhibition when the concentration of [Au(d2pype)2]Cl was higher than 0.5 μM (Figure 1F).

### 3.3. [Au(d2pype)2]Cl Induces Cell Death via Apoptosis in Lymphoma Cell Lines

**Figure 2:**
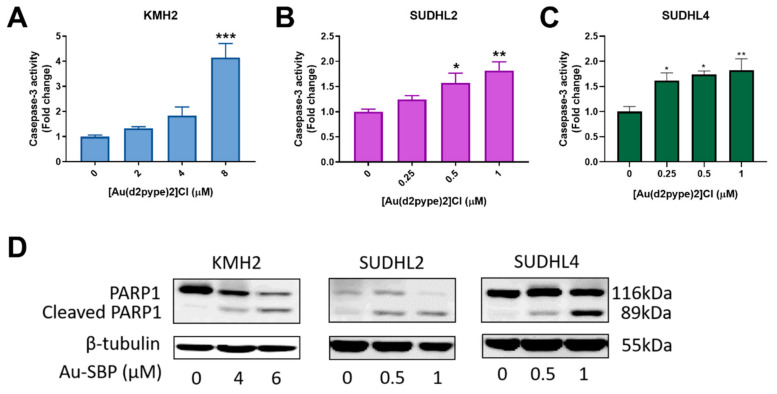
[Au(d2pype)2]Cl induces cell apoptosis in lymphoma cell lines. (**A**) KMH2, (**B**) SUDHL2, and (**C**) SUDHL4 cells were treated with indicated concentrations of [Au(d2pype)2]Cl for 24 h. Caspase-3 activity was measured using a fluorogenic assay. (**D**) The three cell lines were treated with the indicated concentrations of [Au(d2pype)2]Cl for 24 h. Expression of PARP1 and cleaved PARP1 was detected by western blotting. β-tubulin was used as a loading control. Caspase-3 activity was analysed by one-way ANOVA using Dunnett’s post-test. Values indicate mean ± SEM (*n* = 3). * *p* < 0.05, ** *p* < 0.01, *** *p* < 0.001.

Caspase-3 participates in both intrinsic and extrinsic apoptosis pathways in cells. Hence, the activity of caspase-3 can indicate cell apoptosis. The three lymphoma cell lines showed increased caspase-3 activity after treatment with the indicated concentrations of [Au(d2pype)2]Cl. A significant 4-fold increase of caspase-3 activity in KMH2 cells was observed after exposure to 8 μM [Au(d2pype)2]Cl (Figure 2A). In SUDHL2 and SUDHL4 cells, an approximate 1.5-fold increase was obtained at 0.5 μM and 0.25 μM of [Au(d2pype)2]Cl respectively after 24 h treatment (Figure 2B,C). These results showed that caspase-3 activity was significantly increased in lymphoma cells after [Au(d2pype)2]Cl treatment. In addition, the level of cleaved poly[ADP-ribose] polymerase-1 (PARP1), a marker of cells undergoing apoptosis, was increased in all three lymphoma cell lines after [Au(d2pype)2]Cl treatment (Figure 2D). These results indicate that [Au(d2pype)2]Cl causes cell death via caspase-3 apoptosis in lymphoma cell lines.

### 3.4. [Au(d2pype)2]Cl Inhibits Selenoproteins Activity

**Figure 3:**
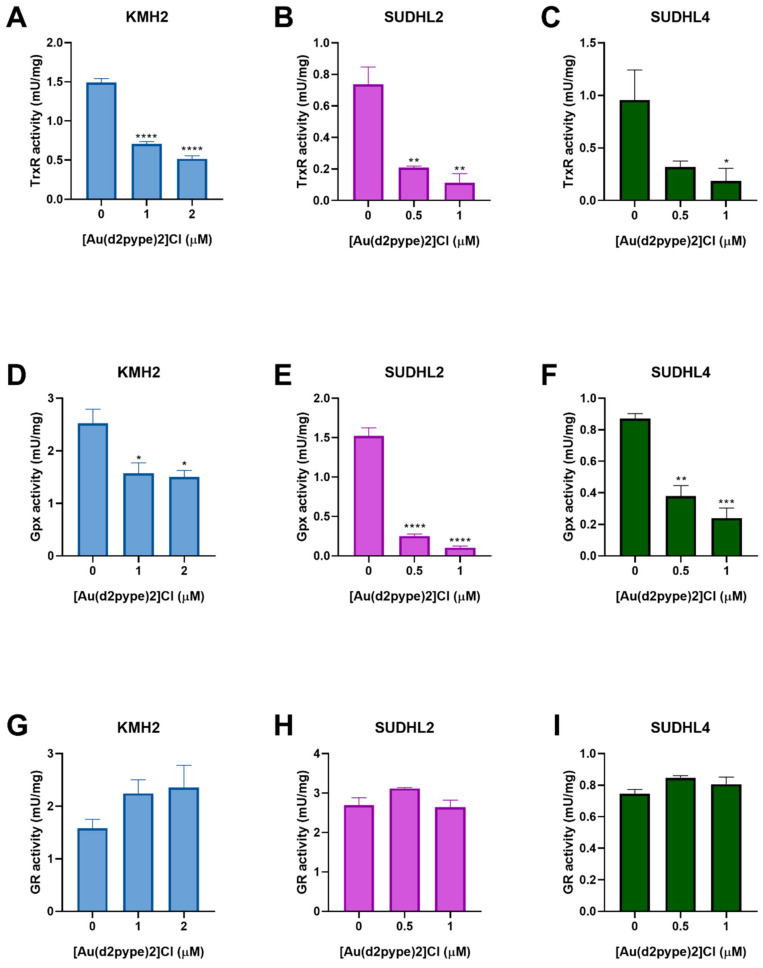
[Au(d2pype)2]Cl inhibits the selenoprotein activity in lymphoma cells. KMH2, SUDHL2, and SUDHL4 cells were treated with the indicated concentrations of [Au(d2pype)2]Cl for 24 h. Protein lysates were prepared and TrxR activity (**A**–**C**), Gpx activity (**D**–**F**), GR activity (**G**–**I**) was measured. Results were analysed by one-way ANOVA using Dunnett’s post-test. Values indicate mean ± SEM (*n* = 3). * *p* < 0.05, ** *p* < 0.01, *** *p* < 0.001, **** *p* < 0.0001.

The effect of [Au(d2pype)2]Cl on antioxidant system activity in lymphoma cells has not been previously tested. Hence, the Trx and GSH systems were assessed for their activity after [Au(d2pype)2]Cl treatment. The result of the TrxR activity assays showed that [Au(d2pype)2]Cl significantly inhibited the activity of TrxR in lymphoma cell lines (Figure 3A–C). In KMH2 cells, treatment with 1 μM [Au(d2pype)2]Cl resulted in a significant decrease of TrxR activity by almost 50% (Figure 3A). In SUDHL2 and SUDHL4 cells, treatment with 0.5 μM [Au(d2pype)2]Cl significantly inhibited TrxR activity by more than 50% (Figure 3B,C).

Glutathione peroxidase (Gpx) and glutathione reductase (GR) are members of the GSH system. Gpx activity assays showed that [Au(d2pype)2]Cl can also inhibit Gpx activity in lymphoma cell lines. In KMH2 cells, treatment with 1 μM [Au(d2pype)2]Cl led to a significant inhibition of Gpx activity (Figure 3D). In SUDHL2 and SUDHL4 cells, a significant decrease of GPx activity was observed when they were treated with 0.5 μM [Au(d2pype)2]Cl (Figure 3E,F). However, GR activity showed no significant inhibition after [Au(d2pype)2]Cl treatment in any cell line. These results suggest that [Au(d2pype)2]Cl can both target TrxR and Gpx in lymphoma cell lines, while not exerting any effect on GR.

### 3.5. Cell Death Is Activated via [Au(d2pype)2]Cl-Induced Oxidative Stress

**Figure 4:**
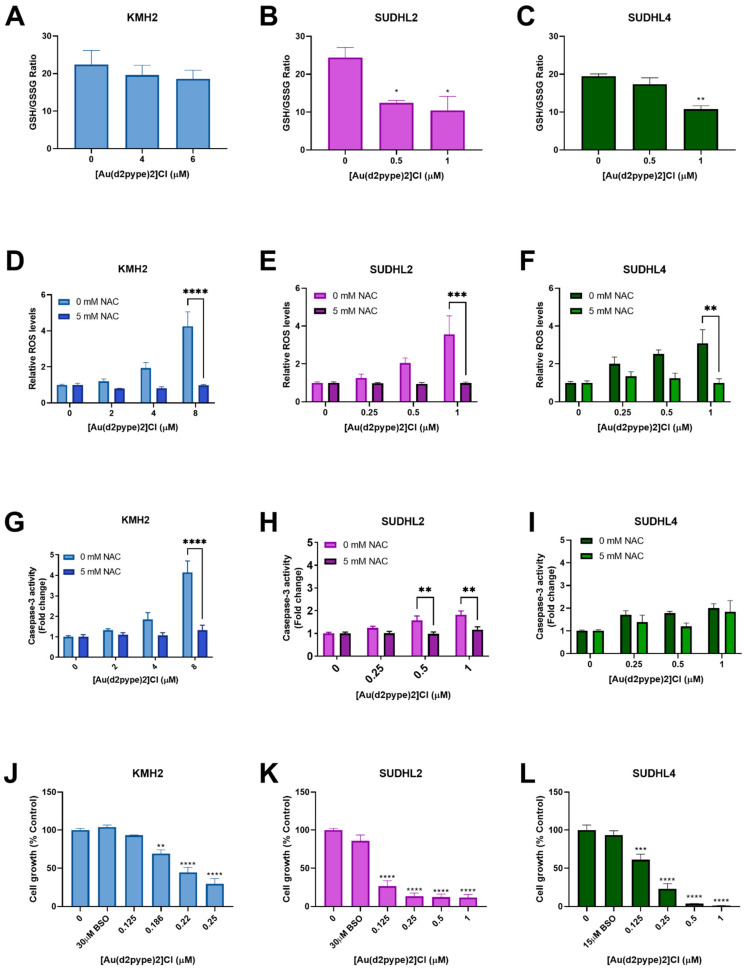
[Au(d2pype)2]Cl generates intracellular ROS and decreases the GSH/GSSG (reduced form of GSH/oxidized form of GSH) ratio in lymphoma cells. KMH2, SUDHL2, and SUDHL4 were treated with [Au(d2pype)2]Cl or co-treated with [Au(d2pype)2]Cl and NAC for 24 h. (**A**–**C**) GSH/GSSG ratio were measured using DTNB reduction. (**D**–**F**) ROS levels were measured using H_2_DCFDA oxidation. (**G**–**I**) Caspase-3 activity was measured using a fluorogenic assay. (**J**–**L**) Cell growth was detected by MTT assay. Results were analysed by one-way ANOVA using Dunnett’s post-test (**A**–**C**,**J**–**L**). Results were analysed by two-way ANOVA using Sidak’s multiple comparisons test (**D**–**I**). Values indicate mean ± SEM (*n* = 3). * *p* < 0.05, ** *p* < 0.01, *** *p* < 0.001, **** *p* <0.0001, compared to untreated cells (**A**–**C**,**J**–**L**), or comparing 0 mM NAC to 5 mM NAC samples (**D**–**I**).

The GSH/GSSG ratio is regarded as a marker for oxidative stress. Under physiological conditions, the GSH/GSSG ratio is approximately 100:1 in resting cells. However, in oxidative stress models, the ratio can be decreased to 10:1 or less [41,42]. Results showed that the GSH/GSSG ratio was significantly decreased in ABC-DLBCL (SUDHL2) and GCB-DLBCL (SUDHL4) cells treated with 0.5 μM and 1 μM [Au(d2pype)2]Cl, respectively, after 24 h. The ratio was decreased to approximately 10:1 in both SUHDL2 and SUDHL4 cells (Figure 4B,C). However, there was no significant change observed in HL (KMH2) cells after [Au(d2pype)2]Cl treatment (Figure 4A).

Redox homeostasis can be controlled by the Trx and GSH systems, both systems protecting cells from damage caused by high levels of ROS. Fluorescence assays that measure H_2_DCFDA oxidation were carried out to determine the ROS levels in lymphoma cell lines exposed to [Au(d2pype)2]Cl for 24 h. With increasing [Au(d2pype)2]Cl concentrations, the ROS levels increased in lymphoma cell lines (Figure 4D–F). In KMH2 and SUDHL2 cells, the highest concentration of [Au(d2pype)2]Cl (8 μM and 1 μM, respectively) caused statistically significant increased ROS levels, which were approximately 4-fold higher than in untreated cells (Figure 4D,E). In SUDHL4 cells, treatment with 1 μM [Au(d2pype)2]Cl resulted in a 2.5-fold significant increased ROS levels (Figure 4F).

To determine whether the cell apoptosis was caused by ROS accumulation, *N*-acetyl-cysteine (NAC), an antioxidant compound that can quench the ROS in cells, was used. The co-treatment [Au(d2pype)2]Cl and NAC indicated that the ROS levels were restored to untreated levels (Figure 4D–F). Meanwhile, caspase-3 activity was also restored in KMH2 and SUHL2 cells upon NAC co-treatment (Figure 4G,H). Although the caspase-3 activity was slightly increased after co-treatment with NAC in SUHL4 cells, the levels were still lower than that observed after [Au(d2pype)2]Cl treatment alone (Figure 4I).

The cellular GSH pool is regarded as a protector in cells since it can participate in both redox control and detoxification [43,44]. To determine the role of the GSH pool in different lymphomas, BSO, an inhibitor of GSH synthetase, was used to co-treat cells with [Au(d2pype)2]Cl. The results showed that cells were more sensitive to [Au(d2pype)2]Cl in the three lymphoma cell lines when co-treated with BSO (Figure 4J–L). It is notable that KMH2 cells showed increased sensitivity to [Au(d2pype)2]Cl when co-treated with BSO (Figure 1B), since a lower concentration of [Au(d2pype)2]Cl was required to inhibit cell proliferation.

### 3.6. [Au(d2pype)2]Cl Modulates Expression of Several Antioxidant Genes

**Figure 5:**
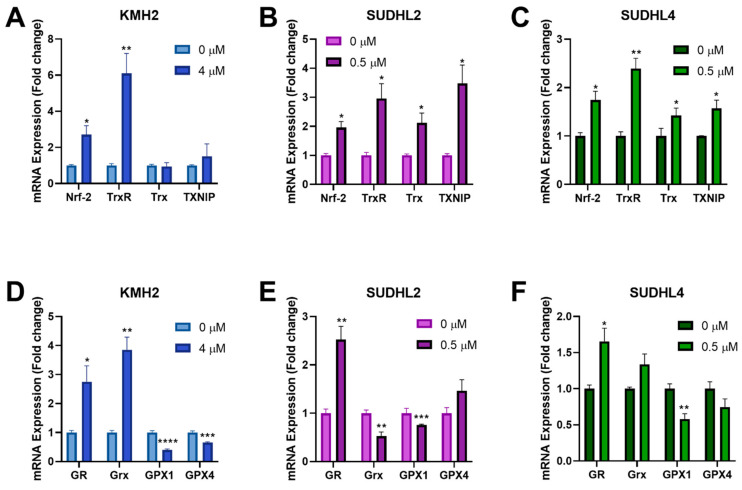
[Au(d2pype)2]Cl modulates the gene expression of antioxidant proteins. (**A**,**D**) KMH2, (**B**,**E**) SUDHL2, and (**C**,**F**) SUDHL4 cells were exposed to the indicated [Au(d2pype)2]Cl for 24 h. mRNA expression levels of Nrf-2, TrxR, Trx, TXNIP, GR, Grx, GPX1, and GPX4 were measured using RT-qPCR. Results were analysed by via two-way ANOVAs with Dunnett’s post hoc test. Values indicate mean ± SEM (*n* = 3). * *p* < 0.05, ** *p* < 0.01, *** *p* < 0.001, **** *p* < 0.0001.

The Nrf-2 transcription factor can be activated by ROS and induces expression of antioxidant genes through binding to the antioxidant response elements in their gene promoters [45]. Since [Au(d2pype)2]Cl was shown to induce higher ROS levels in lymphoma cells (Figure 4D–F), RT-qPCR was performed to determine the effect of [Au(d2pype)2]Cl on Nrf-2 related antioxidant genes response. The results showed that several antioxidant genes were upregulated after [Au(d2pype)2]Cl treatment (Figure 5). In all lymphoma cells, Nrf-2 mRNA expression was significantly increased by approximately 2-fold. In KMH2 cells, analysis of the Trx system gene expression showed that only a significant upregulation of TrxR mRNA was observed (Figure 5A). However, the mRNA level of TrxR, Trx, and TXNIP in SUDHL2 and SUDHL4 cells was statistically significantly increased after [Au(d2pype)2]Cl treatment (Figure 5B,C). With respect to the GSH system, the mRNA level of GR significantly increased, while the mRNA of GPX1 significantly decreased in all lymphoma cells (Figure 5D–F). The Grx mRNA expression also significantly increased in KMH2 cells after [Au(d2pype)2]Cl treatment (Figure 5D); however, a significant decrease was observed in SUDHL2 cells (Figure 5E). The GPX4 mRNA was only significantly decreased in KMH2 cells.

## 4. Discussion

Although many chemotherapies have been developed to cure lymphoma, obstacles such as side effects and drug resistance remain to be overcome. Recently, studies have shown that the microenvironment, including the redox balance, is important for cancer cells to survive and develop [46,47]. In addition, the antioxidant systems are reported to participate in drug resistance mechanisms in cancer cells [48,49,50]. Hence, targeting antioxidant systems may be a possible strategy to overcome drug resistance in lymphoma cells and to offer new opportunities to find new treatments that have less side effects than those caused by the current chemotherapy treatments.

Cancer cells, including lymphoma, have abnormal metabolic activity and proliferation rate. This high metabolic activity leads to high ROS levels, which can damage cancer cells. Therefore, cancer cells with high ROS levels develop the ability to tolerate this microenvironment by increasing the expression or activity of antioxidant systems so that cells can escape from damage. In some cancers, a high expression level of Trx has been recognized as a biomarker [51,52]. Moreover, when compared with normal cells, ROS levels in cancer cells are close to the maximum cytotoxicity threshold. Therefore, targeting antioxidant systems can disrupt the redox balance and push the ROS levels over the cytotoxicity threshold, resulting in cancer death [11,12].

Our laboratory has previously shown that proliferation of both myeloma and chronic myeloid leukemia cells were inhibited by gold-based compounds such as auranofin and [Au(d2pype)2]Cl [30,31]. Although auranofin is considered to be primarily a TrxR inhibitor [53,54], studies have shown that it also targets other proteins, including bcr/abl, NFkB2, or CHORDC1, directly or indirectly [55,56]. [Au(d2pype)2]Cl is another gold-based compound that has been designed to be more selective to cancer cells and to more specifically target TrxR [57]. However, it is likely that it will also bind to and inhibit other selenoproteins [25]. Selenoproteins, which contain a selenocysteine residue, can also participate in thiol/disulfide exchange reactions [58]. Berners-Price et al. have shown that Au(I) phosphine complexes have high thiol reactivity in human plasma [59]. Considering the similar structure and chemical reactions involved between cysteine and selenocysteine [60], proteins containing either cysteine or selenocysteine may be a target of [Au(d2pype)2]Cl. The cytotoxicity of [Au(d2pype)2]Cl has been tested in different cancers, however, it had not been previously tested in lymphoma cells.

In this study, analysis of the Brune lymphoma dataset showed that HL and DLBCL patient samples have higher antioxidant gene expression than that observed in healthy cells. However, it is interesting that not all antioxidants are upregulated in the two types of lymphoma as a general consequence of cancer formation, indicating that the antioxidants with increased gene expression may be integral to the survival or proliferation of lymphoma cells. Other studies have shown that high expression of redox state-regulating enzymes is correlated with a poor prognosis for patients during chemotherapy [61,62]. It was reported that almost all HL patients and a high number of DLBCL patients have high expression of Trx [63] resulting in a poor outcome after cancer chemotherapy. Therefore, targeting the Trx or GSH system may be an effective strategy to eradicate lymphoma cells resistant to current treatments.

In this study, both auranofin and [Au(d2pype)2]Cl significantly inhibited cell proliferation of three lymphoma cells lines: KMH2 (HL), SUDHL2 (ABC-DLBCL), and SUDHL4 (GCB-DLBCL). Results also showed that the growth-inhibiting effectiveness of auranofin and [Au(d2pype)2]Cl depended on the cell line and incubation time. When comparing the three lymphoma cell lines, SUDHL2 and SUDHL4 cells were more sensitive to both auranofin and [Au(d2pype)2]Cl than KMH2 cells. The concentration of [Au(d2pype)2]Cl required to inhibit SUDHL2 and SUDHL4 cell proliferation was approximately 10 times lower than that required to inhibit KMH2 cell proliferation. The SUDHL2 and SUDHL4 cells both showed the same responses in cell proliferation inhibition when treated with auranofin or [Au(d2pype)2]Cl. In the clinic, DLBCL is more aggressive than HL and has poorer patient outcomes [64]. These results indicated that [Au(d2pype)2]Cl has a similar cell growth inhibition activity with auranofin and may be more selective towards aggressive blood cancers. In addition, several studies have shown that the GSH system can compensate for some of the Trx system functions [65,66,67,68,69]. It is therefore possible that the less effective inhibition of KMH2 cells by [Au(d2pype)2]Cl compared to the two other two cell lines (SUDHL2 and SUDHL4) resulted from different basal levels of GSH system activity.

The effectiveness of [Au(d2pype)2]Cl towards the Trx and GSH systems in cells was assessed using antioxidant activity assays. Although the inhibition of the Trx system has been studied in other cancer cells [23], it is the first time that the potential ability of [Au(d2pype)2]Cl to inhibit the Trx and GSH system is determined in lymphomas. [Au(d2pype)2]Cl significantly inhibited both TrxR and Gpx activity, while there was no effect on GR activity in three lymphoma cell lines. TrxR and Gpx are selenoproteins, signifying the presence of a selenocysteine active site amino acid residue, while GR has a cysteine amino acid residue in its active site [70]. Karaca et al. [25] found that gold(I)-based compounds can bind to both selenocysteine and cysteine in proteins, but they target selenocysteine at a lower concentration than that required to target cysteine. This would explain why both TrxR and Gpx were inhibited, but not GR, after treating the cells with the same concentration of [Au(d2pype)2]Cl. In addition, when a significant inhibition of TrxR and Gpx was observed in SUDHL2 and SUDHL4 cells, the cell growth also showed approximately 50% inhibition.

Since TrxR and Gpx both play an important role in regulating ROS [71], the accumulation of ROS in the cells also may reflect the inhibition of those enzymes. In addition, the significantly decreased GSH/GSSG ratio in SUDHL2 and SUDHL4 cells suggests that [Au(d2pype)2]Cl depleted the GSH level in DLBCL. However, the GSH/GSSG ratio in KMH2 cells showed no significant difference after [Au(d2pype)2]Cl treatment. The stable ratio may be one reason to explain why KMH2 cells showed better tolerance to [Au(d2pype)2]Cl, since the GSH pool still remained at a functional level. Our data suggest that [Au(d2pype)2]Cl not only induced ROS generation via TrxR and Gpx inhibition, but also caused GSH depletion, which may be cell specific.

The [Au(d2pype)2]Cl treatment resulted in the activation of the apoptosis pathway, since an increased caspase-3 activity was detected in treated cells. A low level of ROS activates various pathways, including metabolic, inflammatory, and survival pathways [72,73]. However, accumulation of increased ROS will activate apoptosis via various pathways in cells [74]. Moreover, unexpected high ROS in cells can also directly damage DNA and result in cell death. Doxorubicin, a drug widely used in treating lymphoma, is reported to cause oxidative DNA damage [75,76]. However, doxorubicin stimulates both ROS and reactive nitrogen species (RNS) by interacting with nonenzymatic pathways and mitochondrial enzymatic pathways [77], which could cause cardiotoxicity. Thus, new drugs need to be developed to reduce the side effects. To determine whether cell apoptosis is caused by ROS accumulation, NAC, which can quench the excessive ROS, was used. Co-treatment of [Au(d2pype)2]Cl with NAC in HL and DLBCL cell lines showed that ROS levels and caspase-3 activity were restored to normal levels. Although the caspase-3 activity in SUDHL4 cells observed after co-treatment was not significantly decreased to the normal level observed without any treatment, a notable decrease is visible, showing that a similar trend is evident for this cell line. This indicates that [Au(d2pype)2]Cl induced cell apoptosis by an indirect mechanism, which depends on ROS accumulation resulting from inhibition of the TrxR and Gpx selenoproteins.

The GSH system is another essential antioxidant system that protects cells by scavenging ROS via GSH, while the loss of GSH affects cell proliferation [78]. It has been reported that the functional redox homeostasis is essential for cells to survive, and inhibition of GSH synthesis enhances TrxR inhibitors anticancer activity [65,79,80]. Since cancer cells could rely on the GSH system upon loss of functional TrxR [66], Toledano et al. [67] indicated that the GSH system compensates for some functions of the Trx system to maintain cell survival. To investigate whether the GSH pool acts as a protector in lymphoma cells treated with [Au(d2pype)2]Cl, lymphoma cells were co-treated with buthionine sulphoximine (BSO), which is a GSH synthesis inhibitor. Results showed that compared with [Au(d2pype)2]Cl treatment alone, lymphoma cell lines were more sensitive to [Au(d2pype)2]Cl after addition of BSO. It is worth noting that KMH2 cells became extremely sensitive to [Au(d2pype)2]Cl when co-treated with BSO, which indicated the functionality of the basal levels of the GSH system may influence the effectiveness of other potential drug treatments that are targeting the Trx system. Ultimately a patient’s redox system status may form part of an analysis when deciding on a personalized drug treatment regime.

Previous results showed that ROS were accumulated after inhibiting the TrxR and Gpx activity by [Au(d2pype)2]Cl. Since Nrf-2 is a redox-sensitive transcription factor, the mRNA expression of several antioxidant genes regulated by Nrf-2 was studied after [Au(d2pype)2]Cl treatment. This study showed that the Nrf-2 mRNA levels were increased by the ROS accumulation in cells after TrxR inhibition. As Nrf-2 is the main regulator of antioxidant genes, the upregulation of Nrf-2 may affect expression of several antioxidant genes. For the Trx system, the expression of TrxR was significantly upregulated in all three cell lines, while the expression of Trx was only upregulated in SUDHL2 and SUDHL4 cells. With respect to the GSH system, the GR mRNA levels were also increased in all three lymphoma cell lines after [Au(d2pype)2]Cl treatment. It is possible that the GSH system compensates for TrxR to reduce the oxidized Trx after TrxR inhibition [68]. Muri et al. [69] also found that the GSH system compensates for the Trx system functions in developing B cells. It is reported that the Grx utilizes GSH in the process of reactivating Trx by reducing the disulfide bond of Trx. Du et al. [28,68] found that under normal conditions, the GSH system reduced the Trx protein through Grx, and overoxidation of Trx was detected when the TrxR and GSH were inhibited together. In addition, a significantly increased Grx mRNA level was observed in KMH2 cells, while the expression of Grx was decreased or showed no change in SUHL2 and SUDHL4 cells, respectively. This may explain why KMH2 cells showed a better tolerance to [Au(d2pype)2]Cl since there were a large number of GSH system genes upregulated in KMH2 cells. However, the mRNA level of GPX1 and GPX4 decreased by a different degree in all three lymphomas cell lines. Wei et al. [81] and Kinowaki et al. [82] showed that GPX1 and GPX4 overexpression correlated with a poor prognosis in lymphoma patients and also inhibited ROS-induced cell death in vitro. Therefore, the decrease of both GPX1 and GPX4 gene expression indicated that the [Au(d2pype)2]Cl overcomes cell protection based on GPX and induces cell death. In addition, GPX mRNA levels can be regulated by many factors, including the mTOR pathway [83,84], and auranofin has already been found to mediate the inhibition of the mTOR pathway [85]. It is notable that [Au(d2pype)2]Cl and auranofin have many chemical similarities, which raises the possibility that [Au(d2pype)2]Cl may inhibit GPX expression via inhibiting the mTOR pathway. However, further investigation is required. TXNIP was recognized as a tumour suppressor in cells as it can interact with Trx by blocking its biological function [86]. TXNIP acts as a negative regulator of the Trx system by binding to the active site of Trx thereby inhibiting its activity. Researchers found that a low level of TXNIP expression in cancer results in high cancer cell growth [87]. In addition, other studies also showed that TXNIP could directly regulate p53 protein [88], which is a tumour suppressor gene. In this study, increased TXNIP mRNA levels were detected in two DLBCL cell lines, which suggests that the interplay between the Trx system and TXNIP may also be modulated in DLBCL cells after [Au(d2pype)2]Cl treatment.

## 5. Conclusions

In conclusion, inhibiting seleno-antioxidant enzymes using the gold compound [Au(d2pype)2]Cl increased oxidative stress and induced apoptosis in lymphoma cell lines. In this study, we have shown for the first time that the GSH system can also be affected by [Au(d2pype)2]Cl via Gpx inhibition. Moreover, our results in lymphoma cells suggest that it is important to assess the GSH system when inhibiting TrxR function. In addition, the accumulation of ROS activates Nrf-2 mediated gene expression. Taken together, our data indicated that inhibition of TrxR may be potentially applied as part of the current co-treatment regimen for lymphoma.

## Figures and Tables

**Table 1:** List of oligonucleotides.

Target	* Accession Number	Forward	Reverse
L32	NC_000003.12	5′CAGGGTTCGTAGAAGATTCAA GGG 3′	5′CTTGGAGGAAAACATTGTGAGCGATC 3′
Trx	NC_000009.12	5′GC AGTTTATAAAGGGAGAGAGCA 3′	5′TGATCATTTTGCAAGGCCCA 3′
TrxR	NC_000012.12	5′GGAATCCACCCTGTCTCTGC 3′	5′ACGAGCCAGTGG TTTGCAGT 3′
TXNIP	NC_000001.11	5′GGCACCTGTGTCTGCTAAAA 3′	5′CGGGAACATGTATTCTCAAA 3′
GR	NC_000008.11	5′GCTGCTGGCCGAAAACTTG 3′	5′GAATGGCTTCATCTTCCGTGA 3′
Grx	NC_000005.10	5′AACCACACTAACGAGATTCAAGAT 3′	5′AGAGACTAGATCACTGCATCCGC 3′
Gpx1	NC_000003.12	5′CAGTTTGGGCATCAGGAGAAC 3′	5′TCATAAGCGCGGTGGCGT 3′
Gpx4	NC_000019.10	5′AACGTGGCCTCCCAGTGAG 3′	5′GCTTCCCGAACTGGTTACACG 3′
Nrf-2	NC_000002.12	5′GCTCAGTTACACTAGATGAAGAGACA 3′	5′CAGTCATCAAAGTACAAAGCATCT 3′

*—Accession number refers to the gene from which the relevant transcripts are generated.

**Table 2:** Lymphoma cells have up-regulated antioxidant gene expression. mRNA expression in Hodgkin’s lymphoma (HL) and diffuse large B-cell lymphoma (DLBCL) patient samples compared to healthy cells using the Brune lymphoma dataset in Oncomine database [40]. Data was analysed by *t*-test. *n* = 67.

HL			DLBCL		
Gene Name	Fold Change	*p*-Value	Gene Name	Fold Change	*p*-Value
TXN *	4.871	3.31 × 10^−10^	TXN *	3.108	8.72 × 10^−13^
TXN2	1.127	0.003	TXN2	1.126	8.88 × 10^−4^
TXNRD1	1.178	0.004	TXNRD1	1.44	0.02
TXNRD2	1.076	0.127	TXNRD2	1.086	0.01
TXNRD3	1.085	0.059	TXNRD3	1.232	0.011
TXNIP	−1.038	0.659	TXNIP	−1.069	0.75
GPX1 *	5.107	1.12 × 10^−7^	GPX1 *	5.757	4.88 × 10^−8^
GPX2	1.123	0.012	GPX2	1.059	0.11
GPX3	1.423	0.039	GPX3	1.483	0.016
GPX4 *	2.961	8.16 × 10^−6^	GPX4 *	3.642	2.24 × 10^−7^
GPX5	1.027	0.239	GPX5	−1.055	0.898
GPX7	−1.389	1	GPX7	1.481	0.021
GLRX	−1.107	0.726	GLRX	1.388	0.107
GLRX2 *	1.926	7.52 × 10^−6^	GLRX2 *	1.899	6.03 × 10^−6^
GLRX3	1.339	0.0012	GLRX3 *	1.616	1.35 × 10^−6^
GLRX5	1.212	0.121	GLRX5	2.164	9.98 × 10^−6^
GSR	−1.074	0.862	GSR	1.247	0.005
NRE2L2 ^	1.188	0.003	NRE2L2	1.203	1.90 × 10^−4^

*—top 1% upregulated genes (red text). ^—NRE2L2 encodes the Nrf2 protein.

## Data Availability

Data are available from authors on request.
